# Microbial Indicators and Possible Focal Points of Contamination during Production and Processing of Catfish

**DOI:** 10.3390/foods11182778

**Published:** 2022-09-09

**Authors:** Angelica Abdallah-Ruiz, Lurdes Siberio Wood, Taejo Kim, Wes Schilling, Shecoya B. White, Bang-Yuan Chen, Alba Durango-Villadiego, Juan L. Silva

**Affiliations:** 1 Department of Food Science, Nutrition, and Health Promotion, Mississippi State University, Mississippi State, MS 39762, USA; 2 Department of Kinesiology, Health, Food and Nutrition Sciences, University of Wisconsin-Stout, Menomonie, WI 54751, USA; 3 Department of Food Science, Fu Jen Catholic University, 510 Zhongzheng Road, New Taipei City 242, Taiwan; 4 Department of Food Engineering, University of Córdoba, Montería 230002, Córdoba, Colombia

**Keywords:** catfish, *Listeria* spp., *Listeria monocytogenes*, microbial indicators

## Abstract

The catfish industry is important to the United States economy. The present study determined the levels of microbial indicators and the prevalence of *Listeria* spp. and *Listeria monocytogenes* at catfish farms and catfish processing plants. Live fish, water, and sediment samples were analyzed in farms. Fish skin, fillets, chiller water, and environmental surfaces were assessed at the processing plants both during operation and after sanitation. Live fish had 2% prevalence of *Listeria monocytogenes*, while sediment and water were negative for *Listeria*. Live fish skin counts averaged 4.2, 1.9, and 1.3 log CFU/cm^2^ aerobic (APC), total coliform (TCC) and generic *Escherichia coli* counts, respectively. Water and sediment samples averaged 4.8 and 5.8 log CFU/g APC, 1.9 and 2.3 log CFU/g TCC, and 1.0 and 1.6 log CFU/g generic *E. coli* counts, respectively. During operation, *Listeria* prevalence was higher in fillets before (57%) and after (97%) chilling than on fish skin (10%). Process chiller water had higher (*p* ≤ 0.05) APC, TCC, and *Listeria* prevalence than clean chiller water. After sanitation, most sampling points in which *Listeria* spp. were present had high levels of APC (>2.4 log CFU/100 cm^2^). APC combined with *Listeria* spp. could be a good approach to understand microbial contamination in catfish plants.

## 1. Introduction

Farm-raised catfish is the 8th most consumed fish and seafood in the United States [1]. Catfish production primarily includes channel catfish (*Ictalurus punctatus*), blue catfish (*Ictalurus furcatus*), and the hybrid channel catfish female x blue catfish male [2]. The largest producing states are Mississippi, Alabama, and Arkansas with Mississippi as the largest state with respect to production and processing [3].

In the catfish industry, the use of microbial indicators can provide a general assessment of possible fecal contamination, product shelf-life, and sanitation efficacy [4]. This group of microorganisms includes, among others, aerobic bacteria, psychrotrophs, coliforms, and *Escherichia coli* [5,6,7,8]. Aerobic and psychrotrophic counts indicate fish spoilage and inadequate handling during processing [4,9], whereas the presence of coliforms and generic *E. coli* is associated with unsanitary working surfaces, fecal contamination, and possible contamination with pathogens [10,11].

In addition to conventional microbial indicators, *Listeria* spp. could be used as an indicator of a lack of or incomplete sanitation. Prevalence of *Listeria* spp. in catfish processing plants has been reported as 52–85% during operations [12]. Persistent *Listeria* spp. have been related to sites with a lack of or inadequate sanitation and surfaces that are difficult to reach during sanitation, such as surfaces with cracks and drains [13,14]. *Listeria* spp. are ubiquitous in the environment and have unique physiological characteristics. They are a group of Gram-positive bacteria, identified as non-sporulating bacilli and facultative anaerobes. *Listeria* spp. are found in several environments including soil, decaying vegetation, and food processing plants [15,16,17]. The species within the genus can be categorized into two groups: *Listeria sensu strictu*, which includes *Listeria monocytogenes*, *Listeria seeligeri*, *Listeria marthii*, *Listeria ivanovii*, *Listeria welshimeri,* and *Listeria innocua*; and *Listeria sensu lato*, which includes *Listeria grayi* and ten new species discovered since 2009 [18]. This group of bacteria has the ability to grow at refrigeration temperatures (<5 °C), resist high salt concentrations (>10%), and survive at low pH (<5) [19,20].

*Listeria monocytogenes* is recognized as an environmental pathogen and has been isolated from meat, poultry, and seafood processing facilities [21,22,23]. Environmental pathogens are present in the food processing environment and are introduced through ingredients, people, packaging materials, equipment, or any other items entering the facility. They can be transient microorganisms or become persistent in the environment, which can contaminate the food products [24]. *Listeria* spp., including *L. monocytogenes*, have been isolated at different points within catfish processing plants, including food contact surfaces, equipment, utensils, fish skin, and chilled and frozen fillets [12,25,26]. When the processing environment is contaminated, there is a possibility that *Listeria* cells attach to form biofilms on processing surfaces, which increases the risk of contamination in final products [27]. *Listeria* cells can be attached to different surfaces, such as stainless steel, glass, polyethylene, polyurethane, and rubber [28,29]. The bacteria can establish and persist for years even after cleaning and disinfection of the environment [30]. *L. monocytogenes* strains that adhere to surfaces are more resistant to cleaning and disinfection agents than planktonic cells [31,32] since biofilms form more stable niches and are more resistant to sanitizers [33,34].

Since 2016, catfish processing facilities have been required to follow sanitation requirements that were established in the U.S. Department of Agriculture-Food Safety Inspection Service (USDA-FSIS) regulations (Mandatory Inspection of Fish of the Order Siluriformes and Products Derived From Such Fish, 2015) [35]. Indicators of environmental contamination help to identify points in the supply chain that are more vulnerable to microbial contamination in order to establish effective cleaning and sanitation procedures. In addition, microbial levels of live fish at the farm need to be considered to develop strategies to reduce the proliferation of microorganisms during processing, in order to maximize the shelf life of the final products. The total elimination of these microorganisms in the processing environment is a difficult task to accomplish, but through the determination of growth sources, it is possible to identify the sites where cleaning and disinfection is most essential for reducing microbial niches. Studies that have focused on the prevalence of *Listeria* spp. in seafood processing plants have been conducted [12,23,36]. However, the presence of *Listeria* spp. along with other known microbial indicators might help elucidate the most susceptible processing steps in which contamination may occur. Knowing these points in the food chain could help to develop better hygienic practices from farm to fork. Therefore, the first objective of this research was to determine the prevalence of *Listeria* spp. and *Listeria monocytogenes* and the levels of aerobic bacteria, total coliforms, and generic *Escherichia coli* at catfish farms. The second objective was to determine the prevalence of *Listeria* spp. and *Listeria monocytogenes* and the levels of aerobic bacteria, total coliforms, and generic *Escherichia coli* during operation and after sanitation in catfish processing plants.

## 2. Materials and Methods

### 2.1. Sampling Points and Locations

Sampling was conducted in five catfish farms and six catfish processing plants with similar characteristics that were located in the southeastern United States within a 200-mile radius. Catfish farms and processing plants were visited once, having five farm visits and six plant visits in total. Samples taken for testing are described in the first column of Table 1, Table 2, Table 3 and Table 4. At each catfish farm, five ponds were randomly selected and six live fish samples, one water sample, and one sediment sample from each pond were sampled for microbiological analysis during the summer season. At each processing plant, fifteen food contact surfaces (FCS) and five non-food contact surfaces (NFCS) were chosen as sampling points during operation (OP) and after cleaning and sanitation (AS). One of the FCS (fillet chiller) could be sampled only in 4 out of 6 plant visits. In addition, 1 clean chiller water sample, 1 process chiller water sample, 5 fish skin at the receiving station, 5 fish fillets before chilling, and 5 fish fillets after chilling were taken at each plant visit. A total of 538 samples (200 samples from catfish farms and 338 samples from processing plants) were evaluated in this study.

### 2.2. Sample Collection and Sample Preparation

Live fish were sampled by rinsing the fish surface with 225 mL of buffered peptone water (BPW). Environmental surfaces (10 × 10 cm) were swabbed with either sterile cotton swabs containing 1 mL of BPW (Difco, Fisher Scientific, Sparks, MD, USA) for FCS and fish skin or sterile sponge-sticks containing 10 mL of Dey-Engley (DE) neutralizing broth (3M Food Safety, 3M™ Sponge-Stick with 10 mL D/E Neutralizing Broth, St. Paul, MN, USA) for NFCS. Sediment (250 g) and liquid samples (250 mL) were collected in sterile bags and plastic containers, respectively. Fillets were collected in sterile bags. All samples were placed on ice and immediately transported to the Mississippi State University Food Microbiology and Safety Lab at the Department of Food Science, Nutrition and Health Promotion for further analysis. Samples were processed within 4 h after arrival to the lab. The amount of sample portion tested for fillet, water, and sediment was 25 g. This amount was aseptically weighed and individually placed in sterile bags with 225 mL of either buffered *Listeria* enrichment broth (BLEB) for *Listeria* spp. detection or BPW for APC, TCC, and *E. coli* counts. Sediment and fillet samples were homogenized for 1 min in a stomacher (Stomacher 400 Circulator, Seward, Cincinnati, OH, USA). Water samples were hand mixed for 1 min.

### 2.3. Detection and Isolation of Listeria spp.

Samples were enriched and isolated according to the FDA-BAM protocol [37] with some modifications. Buffered *Listeria* enrichment broth (BLEB) was added to the samples prior to incubation for 24 h at 30 °C. Aliquots from turbid samples were streaked on Modified Oxford medium agar plates (Difco, Fisher Scientific) and incubated at 30 °C for 24 h. Non-turbid samples were re-incubated at 30 °C for an additional 24 h. Five presumptive *Listeria* colonies (black colonies surrounded by a halo) were selected from each plate to be confirmed by Multiplex PCR. The selected colonies were transferred to trypticase soy agar (30 °C for 24 h) and then to trypticase soy broth (30 °C for 24 h).

### 2.4. Identification of Listeria spp. by Multiplex PCR

The Multiplex PCR procedure was based on the protocol used by Chen et al. [12] with some modifications. DNA of presumptive isolates was extracted using boiling lysis. First, 1 mL aliquot was transferred to a 1.5 mL microcentrifuge tube and centrifuged at 10,000 rpm × 2 min (Eppendorf, Centrifuge 5415C, Westbury, NY, USA). The obtained supernatant was removed, and the resulting pellet was rehydrated with 50μL distilled water. The sample was then boiled for 5 min and centrifuged again for 2 min. The resulting supernatant was used as the DNA template for Multiple PCR. This technique allowed for the differentiation of *Listeria* spp. into *L. monocytogenes*, *L. inocua*, *L. grayi* and a group of *Listeria seeligeri-Listeria welshimeri-Listeria ivanovii* [38]. The reaction mixture contained (25 μL) 1X Gotaq^®^ Green Master Mix (Promega, Madison, WI, USA), (2 µL) DNA template, four (25 pmol each) forward primers (MonoA [5′-CAAACTGCTAACACAGCTACT-3′], Ino2 [5′-ACTAGCACTCCAGTTGTTAAAC-3′], MugraI [5′-CCAGCAGTTTCTAAACCTGCT-3′], and Siwi2 [5′-TAACTGAGGTAGCGAGCGAA-3′]), and 25 pmol of one reverse primer (Lis1B [5′-TTATACGCGACCGAAGCCAAC-3′]), using the following amplification conditions: 2 min at 98 °C followed by 30 cycles of 30 s at 95 °C, 30 s at 58 °C, 1 min at 72 °C, and a final extension of 5 min at 72 °C [12]. The amplifications were carried out by a thermocycler (Eppendorf, New York, NY, USA). The PCR products were separated by electrophoresis using 1.4% agarose gel and photographed under UV light (BioDoc-itTM Imaging System (UVP, Upland, CA, USA)).

### 2.5. Determination of Aerobic Plate (APC), Total Coliform (TCC), and Generic Escherichia coli Counts

Aerobic plate (APC), total coliform (TCC), and generic *E. coli* counts were determined in all samples using 3M™ Petrifilm™ Plates (3M Food Safety, 3M™ Petrifilm™ Aerobic Count plates, 3M™ Petrifilm™ E. coli/Coliform Count Plates, St. Paul, MN, USA), according to the manufacturer’s instructions. Serial dilutions (10^−1^ to 10^−5^) were carried out for all the samples using 0.1% BPW. An aliquot of 1 mL for each dilution was placed in each petrifilm plate and incubated for 48 h at 35 °C ± 2 °C. After incubation, colonies were counted and reported as log CFU/cm^2^ for live fish and fish skin, log CFU/100 cm^2^ for food contact and non-food contact surfaces, and log CFU/g for fillet, water, and sediment samples.

### 2.6. Experimental Design and Statistical Analysis

For farm samples, a randomized complete block design with three treatments (sample type: live fish skin, pond water, and pond sediment) and five replications (farms as blocks) was utilized to compare APC, TCC, and generic *E. coli* log counts. For overall levels in the processing environment, a randomized complete block design with two treatments (sampling time: during operation and after cleaning and sanitation) and six replications (plants as blocks) was utilized to compare APC, TCC, and generic *E. coli* log counts. For chiller water samples at the processing plants, a randomized complete block design with two treatments (sample type: process chiller water and clean chiller water) and six replications (plants as blocks) was utilized to compare APC, TCC, and generic *E. coli* log counts. For fish samples at the processing plants, a randomized complete block design with three treatments (sample type: fish skin, fillet before chilling, and fillet after chilling) and six replications (plants as blocks) was utilized to compare APC, TCC, and generic *E. coli* log counts. When significant (*p* ≤ 0.05) interactions existed, data were analyzed using Fisher’s Protected least significant difference test to separate treatment means (SAS Version 9.4). In addition, a paired t-test was performed for each sampling point (FCS and NFCS) to find the difference (*p* < 0.05) for APC, coliforms, and *E. coli* log counts between when the plant is in operation and after cleaning and sanitation. The data were reported as mean ± standard deviation (SD) for microbial counts and prevalence for *Listeria* spp. and *Listeria monocytogenes*.

## 3. Results

### 3.1. Indicators in Catfish Pond Samples (Farm)

The presence of *Listeria* spp. (*L. monocytogenes*, *L. inocua*, *L. grayi* or a group of *Listeria seeligeri-Listeria welshimeri-Listeria ivanovii*) and three different microbial indicators (APC, TCC, and generic *E. coli*) were assessed in 150 samples of live fish and 25 samples of both pond water and pond sediment (Table 1). Two percent of live fish (3/150) were positive for *Listeria* spp. that were confirmed as *L. monocytogenes*. Pond water and sediment samples were negative for *Listeria* spp. Pond sediment had the highest (*p* ≤ 0.05) APC counts, followed by pond water and live fish. No significant differences (*p* > 0.05) occurred in TCC and generic *E. coli* counts between live fish, pond water, and sediment (Table 1).

**Table 1:** Aerobic plate count (APC), total coliform count (TCC), generic *E. coli* count, and *Listeria* spp. (Lis) and *Listeria monocytogenes* (Lm) prevalence on live fish skin, pond water, and pond sediment.

Sample Type	APC	TCC	*E. coli*	Lis	Lm
	(log CFU/cm^2^ * or log CFU/g **)			(Positive/Total Samples)	
Live fish skin (LF) *	4.2 ^c^ ± 1.0	1.9 ^a^ ± 1.0	1.3 ^a^ ± 1.0	3/150	3/150
Pond water (PW) **	4.8 ^b^ ± 0.7	1.9 ^a^ ± 2.0	1.0 ^a^ ± 1.9	0/25	0/25
Pond sediment (PS) **	5.8 ^a^ ± 0.8	2.3 ^a^ ± 1.9	1.6 ^a^ ± 1.7	0/25	0/25

* Unit for LF was log CFU/cm^2^; ** Unit for PW and PS was log CFU/g; Limit of detection of APC, TCC, and *E. coli* was 10 CFU/cm^2^ (1 log CFU/cm^2^) for LF and 10 CFU/g (1 log CFU/g) for PW and PS; APC, TCC and *E. coli* results are expressed as mean ± Standard deviation (SD); a,b,c: means with the same letter in the same column are not different (*p* > 0.05).

### 3.2. Indicators in Fish Samples and Water at the Processing Plant

*Listeria* spp. prevalence was 10% (3/30) on fish skin (identified as *L. inocua* and group of *Listeria seeligeri-Listeria welshimeri-Listeria ivanovii*). This prevalence was greater in fillets, with 57% (17/30) and 97% (29/30) in fillets before (BC) and after chilling (AF), respectively. *L. monocytogenes* was not detected on fish skin but was detected on 30% (9/30) and 33% (10/30) of fillets BC and AC, respectively. TCC was greater (*p* ≤ 0.05) in AC fillets than skin and BC fillets, whereas *E. coli* levels were similar (*p* > 0.05) for BC and AC fillets but lower (*p* ≤ 0.05) on fish skin (Table 2).

**Table 2:** Aerobic plate count (APC), total coliform count (TCC), generic *E. coli* count, *Listeria* spp. (Lis) and *Listeria monocytogenes* (Lm) prevalence in the processing environment during operation and after cleaning and sanitation in fish samples and chiller water samples at the processing plant.

Overall Prevalence in the PROCESSING ENVIRONMENT (FCS + NFCS)					
Sampling Time	APC	TCC	*E. coli*	Lis	Lm
	(log CFU/100 cm^2^)			(Positive/Total Samples)	
During operation (OP)	3.6 ^a^ ± 1.6	1.4 ^a^ ± 1.2	<1.0 ^a^ ± 1.0	44/118	25/118
After cleaning and sanitation (AS)	2.1 ^b^ ± 1.6	<1.0^b^ ± 0.9	<1.0 ^b^ ± 0.3	17/118	12/118
**Fish Samples at the Processing Plant**					
**Sample Type**	**APC**	**TCC**	***E. coli***	**Lis**	**Lm**
	(log CFU/cm^2^ * or log CFU/g **)			**(Positive/Total Samples)**	
Fish skin—At receiving (FS) *	3.4 ^a^ ± 0.5	<1.0 ^b^ ± 0.8	<1.0 ^b^ ± 0.4	3/30	0/30
Fillets before chilling (BC) **	3.8 ^a^ ± 1.3	1.2 ^b^ ± 1.3	0.7 ^a^ ± 1.1	17/30	9/30
Fillets after chilling (AC) **	3.4 ^a^ ± 1.8	1.8 ^a^ ± 1.4	0.9 ^a^ ± 1.4	29/30	10/30
**Chiller Water Samples at the Processing Plant**					
**Sample Type**	**APC**	**TCC**	***E. coli***	**Lis**	**Lm**
	**(log CFU/g)**			**(Positive/Total Samples)**	
Process chiller water (PCW)	3.9 ^a^ ± 1.1	1.5 ^a^ ± 0.9	<1.0 ^a^ ± 1.0	5/6	3/6
Clean chiller water (CCW)—before processing	1.1 ^b^ ± 1.3	<1.0 ^b^ ± 0	<1.0 ^a^ ± 0	2/6	0/6

* Unit for FS was log CFU/cm^2^; ** Unit for BC and AC was log CFU/g; FCS: Food contact surfaces; NFCS: Non-food contact surfaces; Limit of detection of APC, TCC, and *E. coli* was 10 CFU/100 cm^2^ (1 log CFU/100 cm^2^) for surfaces (FCS and NFCS) tested OP and AS, 10 CFU/cm^2^ (1 log CFU/cm^2^) for FS, 10 CFU/g (1 log CFU/g) for BC and AC, and 10 CFU/g (1 log CFU/g) for PCW and CCW; APC, TCC, and *E. coli* results are expressed as mean ± standard deviation (SD); a,b: means with the same letter in the same column by sampling time or sample type are not different (*p* > 0.05).

With no significant change in generic *E. coli* load, APC and TCC for chiller water were higher (*p* ≤ 0.05) during processing (process chiller water) than at the start of the day (clean chiller water). In addition, higher *Listeria* spp. prevalence was detected in process chiller water (83%) when compared to clean chiller water (33%) (Table 2). The *Listeria* species that were isolated from chiller water during operation include *L. monocytogenes* (50%) and the group *Listeria seeligeri-Listeria welshimeri-Listeria ivanovii* (33%).

### 3.3. Indicators in Food Contact (FCS) and Non-Food Contact (NFCS) Surfaces at the Processing Plant

Prevalence of *Listeria* spp. and *L. monocytogenes* and the levels of three different microbial indicators (APC, TCC, and generic *E. coli*) were evaluated in 118 environmental samples during operation and after cleaning and sanitation (Table 2). Overall, APC, TCC, and *E. coli* counts were less (*p* ≤ 0.05) after cleaning and sanitation than during operation. Likewise, the prevalence of *Listeria* spp. and *L. monocytogenes* was lower after sanitation (14% and 10%, respectively) than during operation (37% and 21%, respectively) (Table 2). There were 11, 8, and 5 sampling points for APC, TCC, and *E. coli* counts, respectively, that had less (*p* < 0.05) bacteria after sanitation (Table 3 and Table 4).

**Table 3:** Aerobic plate count (APC), total coliform count (TCC), generic *E. coli* count, *Listeria* spp. (Lis), and *Listeria monocytogenes* (Lm) prevalence on food contact surfaces (FCS) during operation (OP) and after cleaning and sanitation (AS).

Sampling Points	Code	APC		TCC		*E. coli*		Lis		Lm	
		(log CFU/100 cm^2^)						(Number of Positive/Total Samples)			
		OP	AS	OP	AS	OP	AS	OP	AS	OP	AS
Deheader	FCS1	4.8 ^a^ ± 0.9	3.2 ^b^ ± 0.6	1.5 ^a^ ± 1.4	1.3 ^a^ ± 1.2	<1.0 ^a^ ± 0.7	<1.0 ^a^ ± 0	3/6	0/6	2/6	0/6
Skinner	FCS2	4.4 ^a^ ± 0.8	3.1 ^b^ ± 1.8	1.7 ^a^ ± 1.1	<1.0 ^a^ ± 1.0	<1.0 ^a^ ± 0.9	<1.0 ^a^ ± 0.7	1/6	1/6	0/6	1/6
Trimming board	FCS3	3.6 ^a^ ± 0.4	1.1 ^b^ ± 1.6	1.4 ^a^ ± 1.3	<1.0 ^b^ ± 0.5	<1.0 ^a^ ± 1.0	<1.0 ^a^ ± 0	2/6	0/6	1/6	0/6
Fillet chiller	FCS4 *	3.3 ^a^ ± 0.2	3.2 ^a^ ± 0.9	1.5 ^a^ ± 1.0	1.8 ^a^ ± 1.2	<1.0 ^a^ ± 0.9	<1.0 ^a^ ± 0	1/4	1/4	¼	0/4
Belt after chiller	FCS5	3.2 ^a^ ± 0.6	<1.0 ^b^ ± 0.7	1.0 ^a^ ± 1.1	<1.0 ^b^ ± 0	<1.0 ^a^ ± 0.7	<1.0 ^a^ ± 0	4/6	0/6	2/6	0/6
Grading table/belt	FCS6	3.3 ^a^ ± 0.3	1.1 ^b^ ± 1.4	1.7 ^a^ ± 0.9	<1.0 ^b^ ± 0.6	<1.0 ^a^ ± 0.8	<1.0 ^a^ ± 0	4/6	0/6	1/6	0/6
Tray in freezer with fish/gray lug	FCS7	2.9 ^a^ ± 0.6	1.7 ^b^ ± 0.9	<1.0 ^a^ ± 0.7	<1.0 ^a^ ± 0	<1.0 ^a^ ± 0.7	<1.0 ^a^ ± 0	1/6	1/6	1/6	0/6
Manual fish conveyor (holding table before skinner)	FCS8	3.5 ^a^ ± 2.8	2.2 ^a^ ± 1.8	2.0 ^a^ ± 1.1	1.1 ^a^ ± 1.3	1.2 ^a^ ± 1.3	<1.0 ^b^ ± 0	0/6	0/6	0/6	0/6
Whole fish skinner	FCS9	4.9 ^a^ ± 1.2	2.0 ^b^ ± 1.5	2.0 ^a^ ± 1.1	<1.0 ^b^ ± 1.1	1.1 ^a^ ± 1.3	<1.0 ^a^ ± 1.1	0/6	0/6	0/6	0/6
Manual trimming table	FCS10	3.6 ^a^ ± 1.9	<1.0 ^b^ ± 1.1	1.8 ^a^ ± 1.2	<1.0 ^b^ ± 0	1.3 ^a^ ± 1.5	<1.0 ^b^ ± 0	3/6	0/6	2/6	0/6
Holding tray (graded fillet)	FCS11	3.6 ^a^ ± 2.2	2.9 ^a^ ± 0.9	2.0 ^a^ ± 1.1	<1.0 ^b^ ± 1.1	1.5 ^a^ ± 0.8	<1.0 ^b^ ± 0	5/6	1/6	2/6	1/6
Belt after chiller tumbler (whole fish)	FCS12	3.7 ^a^ ± 0.7	1.6 ^b^ ± 1.6	1.1 ^a^ ± 1.3	<1.0 ^a^ ± 1.0	<1.0 ^a^ ± 1.1	<1.0 ^a^ ± 0	1/6	0/6	0/6	0/6
Tote with whole fish	FCS13	4.2 ^a^ ± 0.6	2.9 ^a^ ± 1.8	1.5 ^a^ ± 1.2	<1.0 ^a^ ± 0.8	1.0 ^a^ ± 0.9	<1.0 ^b^ ± 0.4	3/6	1/6	3/6	0/6
Belt before injection	FCS14	3.2 ^a^ ± 1.7	1.8 ^b^ ± 1.4	<1.0 ^a^ ± 0.7	<1.0 ^a^ ± 0.9	<1.0 ^a^ ± 0.6	<1.0 ^a^ ± 0	3/6	1/6	2/6	1/6
Injector tank	FCS15	4.1 ^a^ ± 0.6	2.1 ^b^ ± 1.2	1.7 ^a^ ± 1.4	<1.0 ^b^ ± 0.4	1.5 ^a^ ± 0.9	<1.0 ^b^ ± 0	2/6	0/6	1/6	0/6

Limit of detection of APC, TCC, and *E. coli* was 10 CFU/100 cm^2^ (1 log CFU/100 cm^2^) for FCS; APC, TCC and *E. coli* results are expressed as mean ± standard deviation (SD); mean log counts within sampling points with different letters are different by a paired *t*-test (*p* < 0.05); * FCS4 was sampled only in 4 out of 6 plant visits.

During operation, *Listeria* spp. were isolated from 17 (85%) processing points with 15 (75%) isolates identified as *L. monocytogenes*. With respect to other microbial indicators, APC values were above 2.4 log CFU/100 cm^2^ at 18 sampling points (90%), and generic *E. coli* counts were below 2.0 log CFU/100 cm^2^ for all sampling points, regardless of sampling time (Table 3 and Table 4). After sanitation, *Listeria* spp., including *L. monocytogenes,* were not detected in 10 locations (50%). *Listeria* was present at 7 locations. Coliforms and generic *E. coli* were reduced to undetectable levels in 5 (25%) and 16 (80%) locations, respectively. APC were above 2.4 log CFU/100 cm^2^ at 8 points (40%) and reduced below this value at 10 points (50%). Ten environmental samples (FCS: 6; NFCS: 4) were positive for *Listeria* spp. after cleaning and sanitation, including the skinner, fillet chiller, tray in freezer with fish/gray lug, fish holding tray for graded fillets, tote with whole fish, belt before injection, ice container/ice pipe, waste belt, floor, and drain (Table 3 and Table 4).

**Table 4:** Aerobic plate count (APC), total coliform count (TCC), generic *E. coli* count, *Listeria* spp. (Lis) and *Listeria monocytogenes* (Lm) levels on non-food contact surfaces (NFCS) during operation (OP) and after cleaning and sanitation (AS).

Sampling Points	Code	APC		TCC		*E. coli*		Lis		Lm	
		(log CFU/100 cm^2^)						(Number of Positive/Total Samples)			
		OP	AS	OP	AS	OP	AS	OP	AS	OP	AS
Ice container/ice pipe	NFCS1	1.7 ^a^ ± 2.0	1.6 ^a^ ± 1.6	<1.0 ^a^ ± 1.1	<1.0 ^a^ ± 0	<1.0 ^a^ ± 0	<1.0 ^a^ ± 0	2/6	3/6	1/6	3/6
Freezer wall	NFCS2	<1.0 ^a^ ± 1.3	<1.0 ^a^ ± 0.6	<1.0 ^a^ ± 0.6	<1.0 ^a^ ± 0	<1.0 ^a^ ± 0	<1.0 ^a^ ± 0	0/6	0/6	0/6	0/6
Waste belt	NFCS3	4.7 ^a^ ± 1.0	3.3 ^a^ ± 1.8	2.4 ^a^ ± 0.7	<1.0 ^b^ ± 1.0	1.2 ^a^ ± 1.6	<1.0 ^a^ ± 0	1/6	1/6	1/6	1/6
Floor	NFCS4	4.3 ^a^ ± 2.1	3.9 ^a^ ± 0.9	2.0 ^a^ ± 1.6	<1.0 ^a^ ± 1.0	<1.0 ^a^ ± 1.4	<1.0 ^a^ ± 0.5	5/6	3/6	4/6	2/6
Drain	NFCS5	4.6 ^a^ ± 1.2	4.1 ^a^ ± 1.1	1.6 ^a^ ± 1.3	1.2 ^a^ ± 1.0	<1.0 ^a^ ± 1.0	<1.0 ^a^ ± 0	3/6	4/6	1/6	3/6

Limit of detection of APC, TCC, and *E. coli* was 10 CFU/100 cm^2^ (1 log CFU/100 cm^2^) for NFCS; APC, TCC and *E. coli* results are expressed as mean ± standard deviation (SD); mean log counts within sampling points with different letters are different by a paired *t*-test (*p* < 0.05).

## 4. Discussion

In this study, *Listeria* spp. was not found in pond water and sediment and the prevalence was 2% on live fish (Table 1). Low contamination on live fish might be related to the nature of the skin mucus, which is considered the first physical barrier to pathogens in the fish immune system. Fish skin mucus contains antimicrobial substances, such as proteases, antimicrobial peptides, lectins, lysozyme, immunoglobulin, and transferrins [39]. A study by Miettinen and Wirtaneny [40] reported that the number of samples of aquaculture fish contaminated with *Listeria* spp. was typically greater after rainy periods. River waters as well as other runoff waters seemed to be the main contamination source at the farm studied. The farmed fish originally found to carry *L. monocytogenes* become gradually *Listeria*-free (in the pond/farm). Thus, the positive samples could have been from ponds sampled after a rain/runoff event (ponds sampled were filled from water from streams for the most part).

Pond water and sediment had APC ≥ 4.8 log CFU/g and TCC and generic *E. coli* levels ≥1.0 log CFU/g (Table 1). The temperature in summer is commonly greater than 30°C (90°F) in Mississippi, resulting in favorable growth conditions for microorganisms, such as aerobic bacteria, coliforms, and *E. coli*. In addition, fish ponds can accumulate organic matter that comes from animal feed, fecal matter, decaying plankton, and external debris [41]. These sources of nutrients increase the probability that microorganisms survive and grow for long periods in sediment or water. Total coliforms and generic *E. coli* are commonly used as indicators for microbial water quality and fecal contamination [42]. Although these indicators are not used as a direct measure of pathogens, fecal matter may contain foodborne pathogens. Catfish ponds are in an open environment that is exposed to fecal contamination from animals.

At the processing plant, the prevalence of *Listeria* spp. and *Listeria monocytogenes* was low on fish skin and high in fillets after chilling (Table 2). In the present study, a discriminatory typing method, such as whole genome sequencing (WGS), was not performed on the *Listeria* isolates in order to determine a definitive source. However, other authors have found that live fish coming from farm ponds are not a major source of *Listeria* contamination in the processing environment and that the contamination occurs mainly at the processing plant [12,43,44].

Fish skin and fillets, both before and after chilling, had APC and generic *E. coli* levels below the recommended microbiological limits for fresh fish (APC < 5.7 log CFU/g for good quality products and <7.0 log CFU/g for marginally acceptable products, *E. coli* ≤ 1 log CFU/g for good quality products and 2.7 log CFU/g for marginally acceptable products) [9] (Table 2). Removal of heat during chilling from live fish flesh in a relatively short time plus rapid handling during filleting and chilling help maintain the highest possible quality through slowing microbial growth. In addition, APC levels above 7 log CFU/g are commonly reached after 6–7 days of storage [45,46]. Since the catfish were collected and analyzed the same day of processing, the microbial loads were low.

Process chiller water had high APC and TCC counts. These TCC levels are above the EPA Maximum Contaminant Level Goal (MCLG) for drinking water (MCLG = zero mg/L) [47]. Chiller water also had high prevalence of *Listeria* spp. and *L. monocytogenes* (Table 2). The chiller water is recycled and used all day, and this could have caused cross-contamination and the spread of bacteria on some surfaces (Table 3). Moreover, levels of TCC for AC fillets (1.8 log CFU/g) might be associated with cross-contamination from process chiller water since TCC was greater than it was in clean chiller water. Chen et al. [12] identified *L. monocytogenes* in 55.6% (5/9) of chiller water samples, which suggests that this water might be a vehicle for cross-contamination in fresh fillets. Processing live fish to fillets or chilling of fillets was associated with possible contamination.

*Listeria* spp., including *L. monocytogenes*, did not totally disappear on environmental surfaces after sanitation, showing the ability to survive on surfaces that received inadequate/incomplete sanitation. These results are similar to those reported by previous authors [30,36,48]. There were 6 out of 10 positive sites with the presence of *Listeria* spp. after sanitation (tray in freezer with fish/gray lug, fish holding tray for graded fillet, tote with whole fish, belt before injection, ice container/ice pipe, and waste belt) that are made of some type of plastic material (Table 3 and Table 4). Although an attachment strength study for *Listeria* spp. was not performed during this study, different authors have reported that *Listeria* cells can attach better to rubber and plastic than to glass and stainless steel [49,50]. For that reason, it is important to have an effective removal of organic waste and cleaning prior to the application of sanitizers on those sites. In addition, biofilms are very different to remove and can harbor *L. monocytogenes*, making it more difficult to remove this pathogen from processing plants.

*Listeria* spp. and *L. monocytogenes* can survive in the tray in the freezer with fish and in the ice container/ice pipe (Table 3 and Table 4). These bacteria can be isolated from several areas in food processing plants due to their ability to adhere to any contact surfaces at different levels of bacterial attachment. In addition, several authors have considered that non-food contact surfaces are potential sources of contamination with *Listeria* spp. and *L. monocytogenes* in the processing environment and on final food products [12,23]. The drain, floor, and waste belt are points where high loadings of organic waste and water converge from all areas in the food plants.

Seven environmental surfaces were prevalent for *Listeria* spp. after cleaning and sanitation, including the skinner, fillet chiller, fish holding tray with graded fillets, tote with whole fish, waste belt, floor, and drain. *Listeria* prevalence was accompanied with APC levels above the microbiological limits (APC < 2.4 log CFU/100 cm^2^) [51,52]. The high levels of APC on some surfaces might suggest that there was an incomplete removal (inadequate cleaning) of organic matter that was accumulated during processing.

The highest counts of generic *E. coli* were on the fish holding tray and injector tank (Table 3), although they were within the recommended microbiological limits for environmental surfaces (2 log CFU/100 cm^2^) [53]. Montville, Chen, and Schaffner [54] indicated that during food handling, fecal coliforms can be transferred from contaminated hands to food products and subsequently to other surfaces. Reij and Den Aantrekker [55] attributed the incidence of *E. coli* to the lack of hygiene, specifically improper handwashing. Siberio-Pérez [6] reported1.3 and 1.4 log CFU/100 cm^2^ generic *E. coli* on food contact and non-food contact surfaces in catfish plants during processing. This is greater than the counts in this study. *E. coli* was not detected on 7 of the 10 sample sites where *Listeria* spp. was found after cleaning and sanitation. In this study, *E. coli* absence does not indicate the absence of *Listeria*. These results agree with some previous findings, since *E. coli* is an indicator of possible fecal contamination [10], and *Listeria* spp. presence may or may not derive from fecal source [15,16,17,18].

## 5. Conclusions

The data obtained in this investigation indicated that the prevalence of *Listeria* spp. at the farm is low. *Listeria* spp., including *L. monocytogenes,* was not detected in these samples and was low on live fish (2%). APC counts were >4.5 log CFU/g for water and sediment samples and TCC counts >2.0 log CFU/g for sediment. At the processing plant, the prevalence of *Listeria* spp. and levels of indicator microorganisms were higher during processing than after cleaning and sanitation. Once the processing plants were sanitized, there was a lower prevalence of these microorganisms, providing a general idea of how effective and important hygiene and disinfection procedures are at the plant. It is important to state that areas in the processing environment that were positive for non-pathogenic *Listeria* spp. are also considered critical points because they could easily serve as a reservoir for *L. monocytogenes*. In addition, chiller water could be a vehicle of cross-contamination during processing since it contained a higher prevalence of *Listeria* spp., APC, and TCC when compared to clean chiller water. For this reason, it is important to take control measures, such as more frequent water changes, maintaining a low temperature, using antimicrobial treatments, and/or having an adequate water:fish ratio.

## Data Availability

Data are presented within the article.
